# Using average nucleotide identity to improve taxonomic assignments in prokaryotic genomes at the NCBI

**DOI:** 10.1099/ijsem.0.002809

**Published:** 2018-05-24

**Authors:** Stacy Ciufo, Sivakumar Kannan, Shobha Sharma, Azat Badretdin, Karen Clark, Seán Turner, Slava Brover, Conrad L. Schoch, Avi Kimchi, Michael DiCuccio

**Affiliations:** National Center of Biotechnology Information, National Institutes of Health, 9600 Rockville Pike, Bethesda, MD, 20892, USA

**Keywords:** taxonomy, GenBank, RefSeq, type strains

## Abstract

Average nucleotide identity analysis is a useful tool to verify taxonomic identities in prokaryotic genomes, for both complete and draft assemblies. Using optimum threshold ranges appropriate for different prokaryotic taxa, we have reviewed all prokaryotic genome assemblies in GenBank with regard to their taxonomic identity. We present the methods used to make such comparisons, the current status of GenBank verifications, and recent developments in confirming species assignments in new genome submissions.

## Introduction

Approximately 141 000 prokaryotic genomes are currently (March 2018) public in the Assembly database at the National Center for Biotechnology Information (NCBI) and this number is growing exponentially (www.ncbi.nlm.nih.gov/assembly/?term=bacteria). The concurrent increase in data with misassigned taxonomic labels has become a significant challenge. During May 2015 Scott Federhen of the NCBI presented a proposal to address this directly at a workshop attended by a cross section of bacteriologists. A set of broad principles, relying on data from type strains, was proposed and their acceptance marked an important change in how the NCBI curate taxonomic information on prokaryotic genomes [1].

A bacterial type strain is defined in the International Code of Nomenclature of Prokaryotes (ICNP) [2] as the ‘nomenclatural type of the species’ and is the reference point to which all other strains are compared to determine whether they belong to that species. The ICNP requires deposition of declared type strains in at least two independent repositories and these strains are generally well-characterized phenotypically and taxonomically. The annotation of type strains at the NCBI has been available since 2013 when a search term was added to Entrez: ‘sequence from type [filter]’ [3]. This allowed users to constrain searches for sequence records with corresponding type vouchers curated in the NCBI Taxonomy Database [4]. It also opened the possibility to improve the taxonomic annotation of prokaryotic genomes, thus improving microbial resources already available to users at the NCBI [5]. Additionally, genome sequences from type strains are a particularly high-value dataset which until very recently has not been clearly annotated in the NCBI databases. Cooperative projects such as the Genomic Encyclopedia of Bacteria and Archaea (GEBA), which is focused on expanding phylogenetic diversity of prokaryotic genomes and types [6, 7], are rapidly expanding taxonomic coverage for type strain genomes.

Prokaryotic classification has relied on comparisons of genomic similarity since the late 1960s when DNA–DNA hybridization (DDH) was introduced to verify or improve organism clusters via rough genome similarities. Even before genome sequencing was available, it was predicted that complete genome sequence data would be a future standard for determining prokaryotic species [8]. Now that full genome sequences are commonly available, direct comparative methods can accomplish analogous, but more precise results *in silico*. Although several genome-wide similarity statistics are available (e.g. [9]), average nucleotide identity (ANI) is widely used and has been proposed as the best option to determine species boundaries and confirm identification [10, 11]. It also represents a straightforward measure that has proven to have practical scalability for large data sets [12]. Several algorithms already incorporate this measure [13]. Here we present the process by which prokaryotic genomes taxonomy is confirmed or corrected by utilizing type strain genomes at the NCBI.

## Methods

### Type strain selection and verifying the identity of type genomes

Type strain information was extracted from two sources: the NCBI Taxonomy Database (www.ncbi.nlm.nih.gov/taxonomy) and the German Collection of Microorganisms and Cell Cultures at the Leibniz Institute (DSMZ; www.dsmz.de/). Table 1 shows counts of organisms and sequences in these resources.

**Table 1.** No. of type strains (including co-identical strains and other kinds of type materials) from the NCBI Taxonomy Database and the DSMZ

	NCBI taxonomy	DSMZ
No. of organisms	31 078	14 238
No. of type strains (and other type materials)	103 533	45 362

#### NCBI taxonomy database

The NCBI Taxonomy Database is a classification and nomenclature resource for all organisms available in the INSDC public sequence databases and is manually curated by the NCBI Taxonomy group. It serves as a central organizing hub for many NCBI resources as well the two other partners in the International Nucleotide Sequence Database (INSDC) collaboration [14] The database includes type material information (including type strains) sourced from the original publications and other authoritative resources [3] and are linked as attributes of the species.

The NCBI Taxonomy Database records a number of type distinctions based on the three Codes of Nomenclature that governs naming organisms. The current list of accepted type vocabulary approved for use by the NCBI and the INSDC can be found online (www.insdc.org/controlled-vocabulary-typematerial-qualifer). Complete details of the type material identifiers in the NCBI Taxonomy Database along with the corresponding organism names can be obtained from the Taxonomy FTP file ‘names.dmp’ in taxdump.tar.gz (ftp://ftp.ncbi.nlm.nih.gov/pub/taxonomy/).

ICNP-declared prokaryotic type strains are recorded by NCBI taxonomists when the names have been published or can be verified as being ‘in press’ and are associated with sequence records submitted to the INSDC. When duplicate samples of the same original strain are deposited in independent culture collections, these co-identical strains are also indicated as type. Additionally, neotype strains, which are designated by the community after a public review process whenever the original type strain is lost or contaminated, are included.

#### DSMZ

The identification of INSDC genome assemblies from type strains was expanded with additional information retrieved from an outside source. DSMZ provides a frequently updated list of type strains and their associated information as Excel file (www.dsmz.de/support/bacterial-nomenclature-up-to-date-downloads.html). The file was obtained and parsed to extract the type strain names. These were confirmed by NCBI taxonomists and then used to identify type strains for which there is public genome sequence available in the assembly database (available in the online Supplementary Material). The NCBI plans to retrieve information from DSMZ as an ongoing routine activity, to capture new data and corrections to existing data.

#### Refining type strain information

Type strain names from both the NCBI Taxonomy Database and the DSMZ were cleaned to exclude duplicates, partial or incomplete names (e.g. unmatched parentheses or quotes) and to remove any additional text around the names such as the prefix ‘strain’ (e.g. strain wgc123), text in parentheses [e.g. NTCCM0018 (Windhoek)], text in square brackets (e.g. ATCC 29686 [[Halomonas halodurans]]), text following a semi-colon (e.g. ATCC BAA-2439; serovar Sichuan).

#### Type assemblies

The verified list of type strain names was used to seed searches in the GenBank nucleotide database, and all the sequences from type strains were retrieved, and compared to genomic assemblies in the assembly resource. As of March 15, 2018, there are 8008 confirmed type strain genome assemblies in GenBank from 7281 different species, representing 44 % of the ~18 000 described prokaryotic species.

Besides the use of ‘type strain’ and ‘neotype strain’ as defined under the ICNP, two additional terms outside of formal usage were defined to distinguish different classes of reference genomes. These type designations were assigned directly to an assembly and not to sequences:

Assemblies whose sequences were not type strains, but show high matches to marker type sequences (such as 16S sequences) were occasionally assigned with a label defined by the NCBI: ‘proxytype assembly’. A proxytype assembly is designated only if the species has no better type assembly, and the match to marker type sequences is unambiguous and high quality.

A ‘reftype’ is an NCBI-defined term for species which do not have a type culture available and for which a neotype is not likely to be assigned, e.g. species which cannot be maintained in pure culture such as endosymbionts, *Candidatus* organisms, etc. These do not meet the criteria for a type culture but typically have a strain which is considered an *a priori* standard (for example the strain first described for the species). These are flagged as reftype and are only used as a basis for comparison.

Some type assemblies are of low quality (e.g. partial or contaminated). Because the process described here relies heavily on correctly identified set of type assemblies, it is important to exclude these. In addition, some might have incorrect taxonomic identification themselves. For this reason, each type assembly received by GenBank is first evaluated for taxonomic accuracy and contamination using the methods described in this paper. Only the set of confirmed type assemblies is used to analyse other genome submissions.

### Calculation of ANI and determining taxonomic misassignments

#### Analysis

To support proper identification and classification of existing public genome assemblies, the NCBI compares all assemblies against all type assemblies as a routine consistency check. There are two general processes that the NCBI has developed to support this task: a pre-submission process that checks genome submissions to identify any evidence of misidentification or contamination; and a post-submission, global process that executes against all submitted genomes, evaluating matches against all known type-assemblies. The two processes are congruent in terms of the scope of types evaluated, the processing steps performed, and the information reported.

**Fig. 1.**
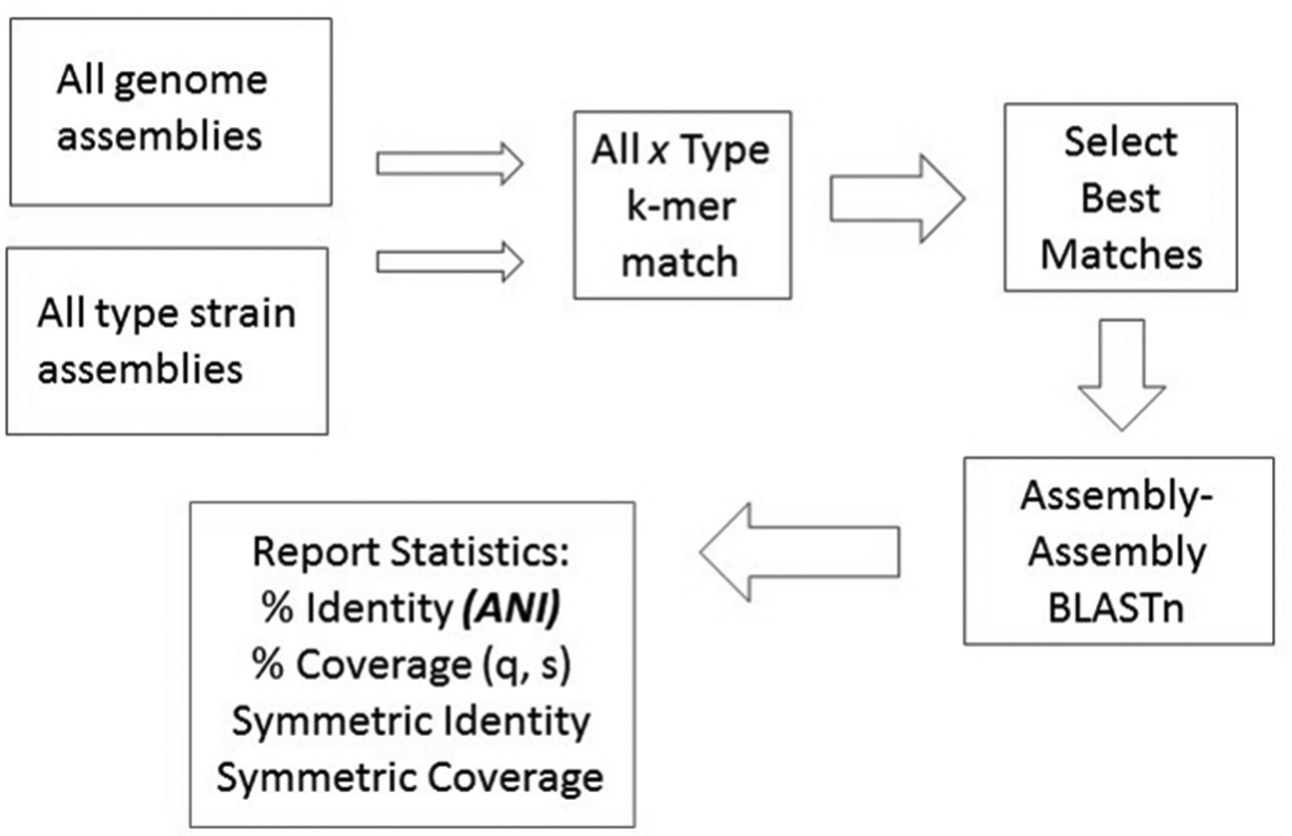
ANI process workflow for processing of pre-submission genomes.

Both processes share a common core set of processing steps (Fig. 1). First, incoming genomes are compared to the existing type assemblies using a fast MinHash-based k-mer comparison [15, 16]. The results of this comparison are used to exclude type assemblies with little or no homology to any given incoming genome. The NCBI’s k-mer comparison uses a word size of 18 and accepts 10 000 MinHash signature values using the Fowler/Noll/ Vo or FNV hash function (www.isthe.com/chongo/tech/comp/fnv/index.html). Out of the k-mer comparison, the NCBI always accepts at least the 40 best-matching type assemblies, and all type assemblies whose MinHash Jaccard distance score is less than 0.995. These parameters were tuned to assure that all matches with an ANI value of at least 80 % would be accepted. In all cases, we assure that we test candidate assemblies against the type assemblies for the declared name of the candidate. For example, given a submission of a *Staphylococcus aureus* genome, we will compare it against: (1) 40 type assemblies which best matched this submission using the k-mer distances; and (2) all type assemblies for *Staphylococcus aureus*, even if the k-mer comparison shows limited or no match.

The k-mer analysis above identifies a set of candidate matches, represented as tuples of query assembly and type assembly. We next evaluate each tuple using pairwise MegaBLAST (specifically, using a word size of 28 and a window size of 150, with gap-affine parameters (-gapextend 1 -gapopen 2) designed to maximize ungapped alignments). These blast alignments are then evaluated for reciprocal best hits. Any aligned range in either the query or type that has a match in more than one location is removed. Note that this analysis may result in splitting an aligned range in the initial blast alignments: it is possible that a given aligned region contains a portion of a genome that could align in more than one location. The resulting alignments thus represent the subset of the blast alignments for which we can uniquely and unambiguously map a location on the genome. These final filtered alignments are then evaluated for overall coverage and percent identity. The percent identity of these alignments is declared as the overall genome-to-type-assembly ANI. Coverage values are used to assure minimal coverage requirements for competent matches. These calculations were made using a custom implementation in C++ based on the NCBI C++ toolkit (www.ncbi.nlm.nih.gov/IEB/ToolBox/CPP_DOC/) and is available on request, pending its release on an ftp site with data required for a complete analysis.

#### Determining ANI cutoffs

Initial surveys of ANI relationships between type assemblies supported the use of a 96 % ANI cutoff to define species boundaries. In order to test this further, we evaluated the difference in ANI for all taxa between taxonomy-matching (concordant) and taxonomy-non-matching (discordant) pairs. We identified 335 taxa (Table 2) for which we found at least 10 GenBank assemblies with ANI alignments above 10 % coverage. This wide net was used to catch cases in which anomalous ANI hits could be represented. For these ANI hits, we further subdivided them based on whether the type assembly's taxon agreed with the submitted assembly’s taxon, and labelled these as concordant (taxonomic agreement) or discordant (taxonomic disagreement). In a perfect world, discordant hits should indicate cases of low-value, low-ANI cross-species alignments, whereas concordant hits should represent cases in which we have high-value confirmatory hits.

**Table 2.** Determination of the default cutoff of 96 %, based on current taxa for which we can determine both concordant and discordant ANI values

ANI threshold	Count concordant below	Count discordant above
98	175	7
97	112	9
96	77	9
95	55	12
94	40	16
93	23	22
92	18	31

As a simplified example consider a single, putative *Salmonella enterica* genome that aligns to six *Salmonella enterica* type assemblies at 98 % ANI (average). That same genome is found to have alignments to one type assembly for each of the following species: *Salmonella bongori*, at 86 % ANI; *Escherichia coli*, at 84 % ANI; and *Enterobacter koseri* at 82 % ANI. The average ANI for this putative *Salmonella enterica* genome matched to its concordant species is 98 %; the average ANI to discordant species is 84 %. This relationship for concordant and discordant values can be observed across all taxa: we expect to see a gulf between concordant and discordant average ANI values. More to the point, a viable species-level cutoff should fall between these two values. Values of average concordant ANI falling below this threshold can be seen as false negative matches (i.e. we fail to confirm the correct species); values of discordant ANI above this threshold can be seen as false positives (i.e. we determine an incorrect match).

Evaluating the selected 335 taxa, a clear distinction can be made between concordant and discordant ANI. The average ANI for concordant pairs is 97.1 %, and the average ANI for discordant pairs is 86.3 %. These data can be further evaluated to test assumptions of the ANI threshold for general species identification. The table below lists the counts of species whose average concordant and discordant ANI values fall below or above a specific ANI cutoff (Table 2).

Given the reported species cutoffs of 94–96 % in the literature (e.g. [11, 17]), and our observations of current taxa for which we can develop both concordant and discordant ANI values, we chose 96 % as our ANI cutoff. This value errs on the side of caution when attempting to make a taxonomic change and minimizes the number of false positives.

We evaluated the competency of matches to determine contamination and misidentification with the following filters. For most taxonomic groups, the assembly is considered a match when the ANI value shows a 96 % identity over 90 % coverage of the genome. For some other taxa, the cutoff range may vary to reflect a clearer or less defined relationship of species within a genus (see examples in Table 3, full list in supplementary material). For example, the acceptance threshold is tuned for known paraphyletic clades (such as *Escherichia coli* and various *Shigella* species); in addition, the system can handle cases in which proper identification requires exquisitely high ANI values, such as between *Mycobacterium africanum*, *Mycobacterium bovis* and *Mycobacterium tuberculosis*. Acceptance criteria can be adjusted to a lower value in cases in which a defined species may be observed to be very broad (e.g. *Acinetobacter baumannii*). In addition, evaluation of coverage statistics can indicate contamination: when coverage values for genome-on-type differ significantly from coverage values of type-on-genome, the suggestion is that the genome being evaluated is either incomplete or may contain a large amount of sequence not accounted for by a particular type assembly.

**Table 3.** Exceptions to ANI cutoff values: for most taxonomic groups, the assembly is considered a match when the ANI value shows a 96 % identity For some taxa, the cutoff range may vary to reflect a clearer or less defined relationship of species within a genus.

TaxID	Scientific name	ANI cutoff
34073	*Variovorax paradoxus*	88.00 %
40324	*Stenotrophomonas maltophilia*	88.50 %
1596	*Lactobacillus gasseri*	93.50 %
	…	
67270	*Streptomyces almquistii*	99.99 %
68178	*Streptomyces avellaneus*	99.99 %
68208	*Streptomyces gibsonii*	99.99 %

#### Processing of new submissions

GenBank currently reviews all new prokaryotic genome submissions for taxonomic accuracy. As part of the standard submission process, microbial genomes are compared with all trusted type genome assemblies. If the genome matches a trusted type assembly of the same species within the defined ANI cutoff, the submission proceeds without further review. If the genome does not match the type of the species, but matches a different type assembly, the report is sent to a curator for manual review. The reviewer considers whether the submission and best-match type assembly are closely related (e.g. the same genus in some cases); whether a type assembly for the presumed name is available for comparison; and the cutoff value for the taxon. The reviewer then decides whether the presumed name should remain as submitted, or whether the submitter should be sent the ANI report for review. In some cases, the submitted genome is clearly in the same genus but does not match any available type assembly, and the submitted name may be changed to a more general name (e.g. *Genus species*). When the ANI review indicates a change should be made, the report is sent to the submitter for their approval before the submission is completes.

For example: A genome submission comes in for *Lactobacillus delbrueckii*, but has a high confidence match to *E. coli*, and a very low match to *L. delbrueckii*. We would flag such a submission for manual review. The result of the review would be to send the ANI report to the submitter before the submission process is completed.

In an average month, 75 prokaryotic genome submissions are sent for manual reviews based on an automated ANI check. Approximately half of these require a taxonomic correction. The majority of the remainder cannot be evaluated sufficiently because the required type strain genome sequences are not yet available.

#### Processing of post-submission genomes and procedure for updating genomes

Not all genomes in GenBank are processed by the NCBI submission data-flow; some are accepted from collaborator organizations [the European Nucleotide Archive (ENA) and the DNA Database of Japan (DDBJ)]. Because of this, and because new type assemblies are added to the collection on an ongoing basis, we perform a global ANI check of all assemblies in GenBank (in other words, treating all assemblies as if they are recently submitted).

Results of post-submission assembly ANI matches are stored in a database for evaluation. We store a limited set of the information including ANI values and coverage values for each matching assembly-to-type-assembly tuple. For each such match, we further record a qualitative assessment of the kind of match, indicating whether the match is a confirmed species-level match, a confirmed genus-level match, a mismatch, and whether the match is above or below the expected ANI threshold for the matching type assembly.

Each day the process tests all of the following circumstances: (1) a new genome which is created in GenBank is evaluated against all type assemblies; (2) a new type assembly is evaluated against all other public assemblies. These new results are combined in the database with all prior ANI data to perform a fresh global analysis for each assembly.

**Fig. 2.**
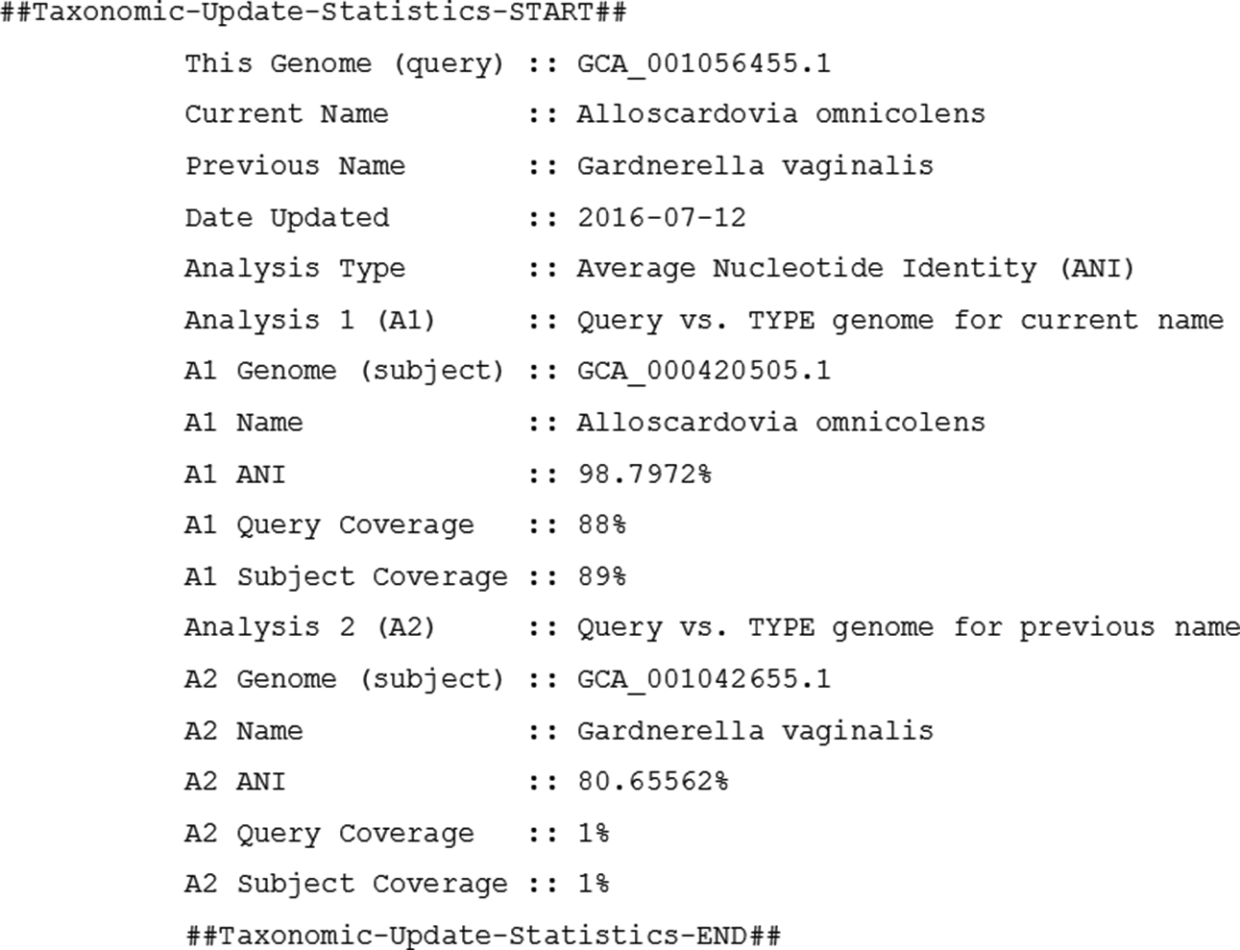
Example of taxonomy correction markup on a GenBank record. This information was added after working closely with the submitter to correct the identification of the genome entry.

When an ANI evaluation shows a clear misidentification, taxonomists manually review each of the proposed changes to confirm they meet the established criteria. GenBank indexers notify the submitter of the proposed change, and allow the submitter 2 weeks to reply with any objections or comments, which are thoroughly reviewed by GenBank staff and taxonomists. After discussion with the submitters, GenBank indexers update the nucleotide records, including records in the BioSample and BioProject databases and genome assemblies. The nucleotides and the assembly will get the Taxonomic-Update-Statistics structured comment in this process (see Fig. 2). In consultation with the other partners in the INSDC, the NCBI now has the authority to change identifications where the ANI process indicates a change is warranted.

#### Special cases

In addition to tuning ANI thresholds for acceptance of species-level matches, invariably there are some difficult-to-evaluate cases that are either unresolvable based purely on ANI or that require further clarification in the literature. The NCBI can handle such cases through the development of an internally mapped set of ANI equivalency groups (Table 4). These equivalency groups can be used to automatically pass certain difficult to resolve groups, such as *Bacillus cereus* and *Bacillus anthracis*, proper separation of which requires evaluation for the presence of specific genes.

**Table 4.** ANI equivalency groups Pairs of species which cannot be separated by ANI analysis due to high similarity of their genome sequences. Sometime, an equivalency group will include more than two species. In these cases, they are duplicated in the lookup table.

Species_1	Species_2
*Brucella ovis*	*Brucella melitensis*
*Bacillus cereus*	*Bacillus anthracis*
*Bacillus thuringiensis*	*Bacillus anthracis*
*Brucella neotomae*	*Brucella melitensis*
*Brucella suis*	*Brucella melitensis*
*Brucella canis*	*Brucella melitensis*
*Bacillus velezensis*	*Bacillus amyloliquefaciens*

**Fig. 3.**
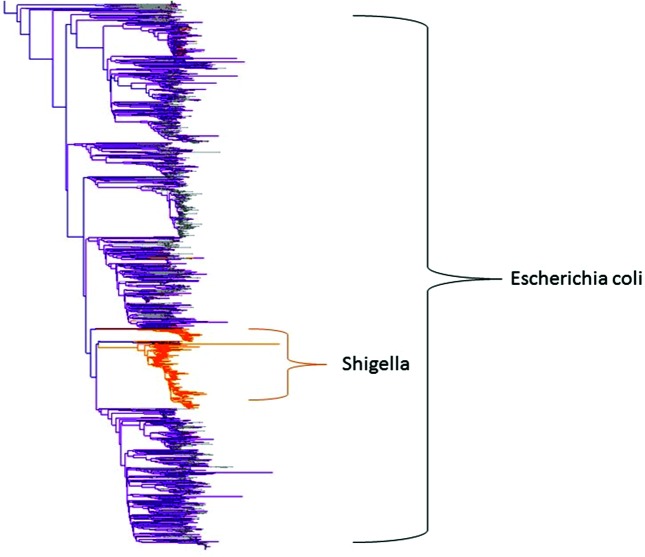
K-mer tree showing the distribution of *Shigella* genomes among those of *E. coli.* The bar indicates the percentage nucleotide rate over the length of the genome.

In certain cases, while we have the discriminatory power to discern differences between some species, for historical reasons it would not serve the purposes of the community to make a change. Such is the case, for example, with *E. coli* and various *Shigella* species (Fig. 3). Our system automatically ignores a small number of such cases, treating the matches as loosely defined equivalents. While we strive to present a truthful and accurate accounting of each genome, the goal of the system is to preserve the historical assignment where there is community consensus that such an assignment is worthwhile.

#### Determination of cutoff values

Some genera contain species which, when grouped at 96 % identity, do not form groups that reflect the species boundaries. These taxa are given cutoff values which more accurately reflect their species assignments. The list of species with non-standard cutoff points is provided in the supplementary material.

**Fig. 4.**
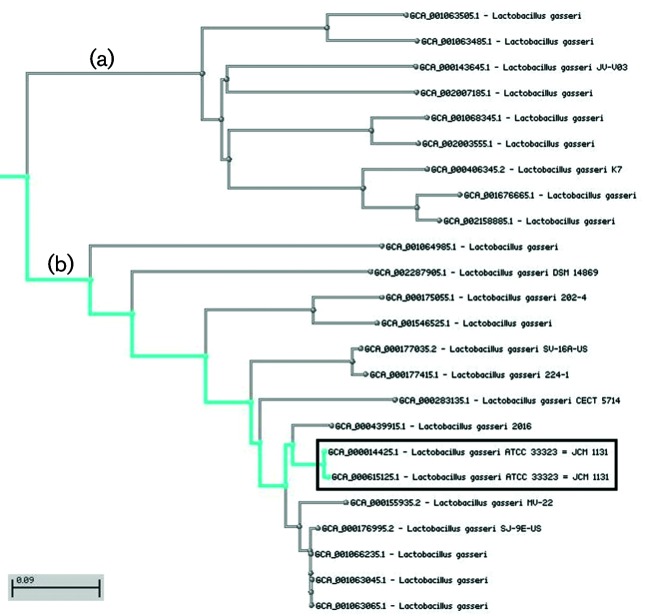
K-mer tree showing genome variability amongst *Lactobacillus gasseri* assemblies. The ANI cutoff of 93.5 % includes both groups of assemblies (a and b), whilst a 96 % cutoff will separate them. Type assemblies are highlighted. The bar indicates the percentage nucleotide substitution rate over the length of the genome.

We should note that accurate determination of all cutoffs is an area of active research, and ANI threshold processing needs additional effort. We intend to keep adjusting our curation efforts in consultation with the research community to make changes only in situations that are clearly defined. For example, within *Mycobacterium*, we likely should accept as a true match any match to the same species above ANI threshold for that taxon and with very high coverage (i.e. 95 %+coverage). Several genome assemblies that are declared as *Mycobacterium tuberculosis* with an ANI above 99.8 % at 98 % coverage have a better match to the type for *Mycobacterium africanum* – at 99.85 % ANI, sometimes with slightly lower coverage. These matches are not easily distinguishable via ANI, and we do not consider such very close mismatches as true mismatches. In other situations a broader cutoff is necessary. For example, *Lactobacillus gasseri* is most efficiently grouped with a cutoff value of 93.5 %, based on the submitter identifications. A k-mer distance tree (Fig. 4) indicates that 2 groups can be distinguished within this current cutoff. The assemblies above node B are closely aligned with the type assemblies for the species, while another group of assemblies above node A appear to be more distant. A cutoff of 96 % would account for separating both groups and it is possible that the assemblies above node B represents an undescribed species. However, in this and other similar situations we will continue to use the expanded cutoff to account for broader species concepts until new taxonomic information (with associated type assemblies) allow us to adjust. Once a process is in place, our intention is to release these updated values as part of the periodic taxonomy ftp releases (ftp://ftp.ncbi.nlm.nih.gov/pub/taxonomy/).

## Results and discussion

Our current application of ANI relationships between type assemblies resulted in the following:

- Using a default cutoff range of 96 % identity and 90 % coverage, species identities were tested via pairwise ANI comparison to determine whether the suggested range could be applied to all species within a genus or family.
- There was an evident distinction between intra-and inter-species relationships at 96 % for most taxonomic groups. This cutoff confirms previously published information [11, 17]. This value is conservative and attempts to avoid making any inappropriate changes in edge cases.
- Exceptions to this were genera that were unusually highly related or have unusual or undescribed diversity. There are approximately two dozen taxa having a divergent threshold used for identification (see the supplementary material.)

New submissions of prokaryotic genomes into GenBank are screened using ANI to known type assemblies. In cases where new submissions are positive matches (96 %, or cutoff previously defined for the taxon), the submission proceeds without a taxonomy consult.

Of the 141 000 prokaryotic genome assemblies in GenBank, approximately 66.8 % can be confirmed as correctly identified by comparison with confirmed type strains using ANI methods. Approximately 3.6 % can be confirmed as misidentified. The remainder (29.6 %) generally cannot be evaluated due to a lack of relevant type strain assemblies. To date the ANI process has enabled the correction of approximately 750 previously submitted genomes.

### GenBank global genome species verification

We have developed a process which uses the ANI method to improve the accuracy of taxonomic identification of sequenced prokaryotic genomes. This method is used to assess new submissions to GenBank on a daily basis, and to notify submitters if there appears to be a misidentification. Genomes which have already been made public in GenBank have been evaluated against all confirmed type assemblies, and this evaluation process is repeated whenever a new type assembly is submitted and confirmed.

Future work will use a similar method to identify areas of contamination in both public and newly submitted genomes. This information will be communicated to the submitters along with suggested cleanup methods. This cleanup can also be performed by the NCBI, followed by a reannotation for GenBank/Refseq.

In many cases, the full genome sequence of the type strain is not available, but 16S gene and other conserved markers are available. In these cases, the marker sequences can be used to evaluate existing strains of the same species to identify a possible ‘proxytype’, or stand-in for the type or neotype until one becomes available. The utility of using this method remains under evaluation.

Additional future work will involve development expanding this process to eukaryotes. The eukaryotic process will start with fungi with particular focus on yeasts (single cellular fungi occurring in several disparate lineages). This group has the benefit of relatively small genome sizes, with a number of well-assembled genomes for evaluation, and a diverse taxonomic range which can be used to recalculate parameters. The development of the process for yeasts will be followed by other fungi, then invertebrates with small genomes, and finally to other eukaryotes. The eukaryotic process will be used for both taxonomic identification and removal of contaminants from genome sequence.

## Supplementary Data

397 Supplementary File 1
