# Supplementary material for: Using average nucleotide identity to improve taxonomic assignments in prokaryotic genomes at the NCBI
Source: Int J Syst Evol Microbiol. 2018 May 24;68(7):2386–92. doi: 10.1099/ijsem.0.002809 (PMC6978984; doi:10.1099/ijsem.0.002809)
Supplement: Supplementary File 1 [file ijsem-68-2386-s001.pdf]

scientific_name	species_taxid	count_concordant	ani_concordant	count_discordant	ani_discordant	ani_diff
Borreliella burgdorferi	139	98	98.90816542	46	91.06390144	7.844263981
Brachyspira hyodysenteriae	159	26	99.13750804	16	87.19409736	11.94341068
Leptospira interrogans	173	278	99.29517665	163	84.99151384	14.3036628
Leptospira borgpetersenii	174	35	99.15387857	406	84.03191096	15.12196761
Azospirillum brasilense	192	10	97.43933996	10	83.97283936	13.4665006
Campylobacter coli	195	810	97.63563698	1209	85.56741231	12.06822466
Campylobacter fetus	196	64	93.32570994	22	82.69987476	10.62583517
Campylobacter jejuni	197	1160	97.22506746	881	85.49048804	11.73457942
Campylobacter hyointestinalis	198	19	98.35215491	67	86.054786	12.29736891
Campylobacter lari	201	10	94.87756348	1409	80.97513149	13.90243198
Helicobacter pylori	210	857	94.67585887	12	86.78959052	7.886268349
Helicobacter felis	214	21	97.16886902	17	83.06363992	14.1052291
Brucella abortus	235	183	99.92975236	365	97.4508235	2.478928856
Brucella ovis	236	15	99.98727671	532	98.0677314	1.919545315
Elizabethkingia meningoseptica	238	21	99.05593243	89	82.88523998	16.17069245
Francisella tularensis	263	227	98.53462342	30	84.80945347	13.72516995
Comamonas testosteroni	285	26	94.33528534	78	84.13075022	10.20453512
Pseudomonas aeruginosa	287	2627	99.21002183	1017	83.78916586	15.42085597
Burkholderia cepacia	292	97	97.07458191	1919	87.62910168	9.445480236
Pseudomonas fluorescens	294	117	86.71377723	3860	82.70291638	4.010860851
Pseudomonas fragi	296	20	93.53100345	1190	83.19371126	10.33729219
Pseudomonas putida	303	85	89.42169908	3062	82.53160613	6.89009294
Ralstonia solanacearum	305	77	95.0166946	70	84.31648058	10.70021402
Pseudomonas stutzeri	316	47	89.86598871	2579	82.61552558	7.250463129
Pseudomonas syringae	317	279	90.66846934	348	86.09038263	4.57808671
Ralstonia pickettii	329	12	94.37283603	110	85.59922489	8.773611138
Xanthomonas campestris	339	67	91.01320203	983	86.88792624	4.12527579
Xanthomonas translucens	343	45	96.92693498	1049	83.68248133	13.24445365
Xanthomonas citri	346	103	98.24404801	908	88.36647689	9.877571119
Xanthomonas oryzae	347	153	99.33454312	747	87.24899708	12.08554604
Agrobacterium tumefaciens	358	33	91.59790595	81	87.70588668	3.892019278
Bradyrhizobium japonicum	375	23	94.62413666	162	86.21770037	8.406436295
Sinorhizobium fredii	380	14	96.66170829	284	83.23642873	13.42527956
Rhizobium leguminosarum	384	43	93.34127789	236	84.8578678	8.483410092
Acetobacter pasteurianus	438	22	94.43856448	25	84.5046069	9.933957579
Gluconobacter oxydans	442	13	91.34567848	25	82.54647705	8.799201425
Acinetobacter baumannii	470	2484	97.92873927	416	87.15091818	10.7778211
Acinetobacter calcoaceticus	471	16	94.78274265	2866	86.99183929	7.790903369
Neisseria gonorrhoeae	485	441	99.45768042	1479	94.3268164	5.130864022
Neisseria lactamica	486	11	93.65658951	1909	93.02617855	0.630410963
Neisseria meningitidis	487	1367	97.4094772	553	93.67654084	3.732936363
Bordetella bronchiseptica	518	70	98.90853795	824	95.17953505	3.729002895
Bordetella pertussis	520	627	99.8676106	266	88.40585577	11.46175483
Chromobacterium violaceum	536	16	97.26221752	45	85.66414235	11.59807517
Eikenella corrodens	539	11	97.2084137	29	89.44843384	7.759979853
Citrobacter koseri	545	12	99.07762718	24717	83.96903229	15.10859489
Citrobacter freundii	546	101	97.70780357	19276	83.3037263	14.40407728
Klebsiella aerogenes	548	135	98.44498229	4258	86.0678885	12.37709379
Pantoea agglomerans	549	21	97.02606092	175	83.03338858	13.99267234
Enterobacter cloacae	550	572	88.74315146	19858	83.09237392	5.65077754
Erwinia amylovora	552	34	99.88409008	34	83.41113019	16.47295989
Pantoea ananatis	553	39	97.76255688	119	82.180724	15.58183288
Pectobacterium carotovorum	554	66	93.98625364	78	84.66435227	9.321901368
Escherichia coli	562	10022	97.69048085	13864	84.83541672	12.85506413
Hafnia alvei	569	10	93.7203804	10	87.63568268	6.084697723
Klebsiella oxytoca	571	106	95.51431598	15029	83.22829553	12.28602045
Klebsiella pneumoniae	573	3515	99.07311748	21186	83.49987665	15.57324084
Raoultella planticola	575	11	99.43074452	5312	84.28038935	15.15035516
Proteus mirabilis	584	70	98.81720472	14	85.00992966	13.80727506
Providencia stuartii	588	13	98.21937854	26	82.59956154	15.619817
Serratia marcescens	615	349	95.78906058	205	85.62929989	10.15976069
Shigella boydii	621	51	98.98327884	19201	92.01911873	6.964160104
Shigella flexneri	623	82	99.82786095	19154	91.89022583	7.937635117
Shigella sonnei	624	999	99.81459494	18238	91.64585022	8.168744723
Yersinia enterocolitica	630	174	97.30331033	526	84.93525871	12.36805162
Yersinia intermedia	631	19	96.73331612	680	84.89901653	11.83429959
Yersinia pestis	632	300	99.95642749	399	86.4855065	13.47092098
Yersinia pseudotuberculosis	633	49	99.10985861	650	91.36311194	7.746746674
Edwardsiella tarda	636	12	95.04231366	17	84.69096823	10.35134543
Aeromonas hydrophila	644	62	96.14907641	174	87.86865815	8.280418263
Aeromonas salmonicida	645	41	98.62918725	195	86.67925681	11.94993044
Aeromonas caviae	648	12	98.00749553	224	87.27437736	10.73311817
Aeromonas veronii	654	41	96.38899657	195	87.31860183	9.070394736
Photobacterium angustum	661	13	97.56047704	37	83.62100323	13.93947381
Vibrio alginolyticus	663	36	97.62298518	1107	84.60919583	13.01378935
Vibrio cholerae	666	702	99.04349982	81	84.02715989	15.01633993
Vibrio harveyi	669	35	96.98679071	1268	83.55148813	13.43530258
Vibrio parahaemolyticus	670	797	98.4870231	346	83.29169009	15.19533302

Vibrio vulnificus	672	116	97.61350073	988	82.58982766	15.02367307
Vibrio fluvialis	676	10	98.19464535	799	81.01746627	17.17717908
Vibrio campbellii	680	27	96.8101849	1218	83.6248468	13.1853381
Aggregatibacter actinomycetemcomitans	714	37	97.71753074	11	83.2634312	14.45409954
Haemophilus haemolyticus	726	10	95.79578781	478	90.64877107	5.147016741
Haemophilus influenzae	727	404	97.26700872	82	86.53154066	10.73546805
Haemophilus parainfluenzae	729	24	94.88883624	473	84.74265337	10.14618287
Gallibacterium anatis	750	26	97.43505463	10	85.97076721	11.46428741
Bartonella bacilliformis	774	16	99.22146174	93	81.48912856	17.73233318
Ehrlichia ruminantium	779	10	96.44986513	13	81.95751014	14.49235499
Rickettsia prowazekii	782	11	99.94196042	107	88.37643461	11.56552581
Bartonella quintana	803	12	99.90409342	98	84.10671219	15.79738124
Chlamydia trachomatis	813	144	99.36392154	58	80.9485104	18.41541114
Bacteroides thetaiotaomicron	818	14	97.979599	59	85.13001665	12.84958235
Bacteroides uniformis	820	18	98.51357112	24	85.93709326	12.57647786
Bacteroides vulgatus	821	13	99.33850685	16	93.97356272	5.364944128
Porphyromonas gingivalis	837	59	98.59584679	13	92.53852081	6.057325977
Fusobacterium nucleatum	851	57	93.10029645	35	89.89310147	3.20719498
Fusobacterium periodonticum	860	15	92.7247167	75	87.47761505	5.247101644
Leuconostoc gelidum	1244	11	96.66191826	22	81.51504257	15.14687569
Leuconostoc mesenteroides	1245	52	97.87525363	28	84.76854189	13.10671174
Pediococcus acidilactici	1254	22	98.20329842	14	81.14441554	17.05888288
Pediococcus pentosaceus	1255	12	99.14387258	22	81.40235623	17.74151634
Micrococcus luteus	1270	24	97.6735843	16	91.29054308	6.383041223
Staphylococcus aureus	1280	8649	98.82383125	253	87.96140449	10.86242675
Staphylococcus epidermidis	1282	481	99.3142257	586	83.54344637	15.77077933
Staphylococcus haemolyticus	1283	168	99.16696167	901	83.6102908	15.55667087
Staphylococcus xylosus	1288	18	96.69086499	109	82.94193457	13.74893042
Staphylococcus hominis	1290	49	98.37480693	889	83.77760435	14.59720258
Staphylococcus sciuri	1296	17	98.24975496	14	87.38739341	10.86236156
Streptococcus gordonii	1302	25	95.51467752	142	87.53564282	7.979034704
Streptococcus oralis	1303	85	93.50228455	8310	87.27986693	6.222417617
Streptococcus salivarius	1304	44	95.64141163	79	90.74894177	4.892469859
Streptococcus sanguinis	1305	30	95.98241911	63	87.52673951	8.455679599
Streptococcus thermophilus	1308	44	99.19395742	65	89.86115547	9.33280195
Streptococcus agalactiae	1311	909	99.14002155	29	98.4273779	0.71264365
Streptococcus pneumoniae	1313	8090	98.67169034	277	88.59196063	10.07972971
Streptococcus pyogenes	1314	337	99.02824378	305	84.58476358	14.44348021
Streptococcus parasanguinis	1318	26	95.05034168	1451	85.47274575	9.577595933
Streptococcus anginosus	1328	39	95.27829281	48	91.24314938	4.035143431
Streptococcus dysgalactiae	1334	26	97.56624695	586	86.37700273	11.18924422
Streptococcus equinus	1335	31	93.48268738	38	85.24569391	8.236993468
Streptococcus equi	1336	246	98.37022988	295	84.31951217	14.05071771
Streptococcus intermedius	1338	15	96.99565887	147	86.65935091	10.33630797
Streptococcus parauberis	1348	22	99.13282845	27	82.47485182	16.65797663
Streptococcus uberis	1349	14	98.18179812	41	81.96681232	16.2149858
Enterococcus faecium	1352	928	99.00803326	104	88.48320374	10.52482952
Enterococcus hirae	1354	30	99.40498234	1004	83.42873647	15.97624587
Bacillus amyloliquefaciens	1390	51	95.20960909	362	86.01116503	9.198444059
Bacillus anthracis	1392	206	98.86059689	2069	91.66350309	7.197093794
Bacillus cereus	1396	983	94.43785532	1293	92.65312886	1.784726458
Bacillus licheniformis	1402	67	99.16314535	39	91.31814605	7.844999309
Bacillus megaterium	1404	79	97.14970046	31	95.63240834	1.517292121
Bacillus mycoides	1405	59	95.330369	2217	90.04264888	5.287720114
Paenibacillus polymyxa	1406	30	93.69930115	23	90.04706175	3.652239393
Bacillus pumilus	1408	44	92.68778894	77	91.08326013	1.604528814
Lysinibacillus sphaericus	1421	16	85.88037395	38	83.85025627	2.030117687
Bacillus subtilis	1423	159	96.20147019	257	83.12061663	13.08085356
Bacillus thuringiensis	1428	475	96.56841251	1801	92.3856945	4.182718005
Bacillus atrophaeus	1452	27	98.92330126	387	81.38551626	17.53778499
Bacillus simplex	1478	13	94.03698455	21	91.93104735	2.105937201
Clostridium botulinum	1491	130	97.632039	28	92.95901544	4.673023559
Clostridioides difficile	1496	1170	99.14745249	13	99.12606577	0.021386721
Paeniclostridium sordellii	1505	43	98.20751084	13	83.64697234	14.5605385
Clostridium sporogenes	1509	23	97.28061719	136	92.61898216	4.661635033
Clostridium beijerinckii	1520	19	96.98259494	19	87.18597412	9.796620821
[Clostridium] clostridioforme	1531	16	98.70909678	17	90.39312721	8.315969571
Lactobacillus acidophilus	1579	20	99.9676578	142	82.60129535	17.36636245
Lactobacillus casei	1582	46	85.01960293	194	83.62636771	1.393235226
Lactobacillus helveticus	1587	30	98.82068581	125	83.76398308	15.05670273
Lactobacillus pentosus	1589	12	98.02048747	248	83.19766202	14.82282545
Lactobacillus plantarum	1590	238	98.11473228	25	84.7883815	13.32635078
Lactobacillus gasseri	1596	26	97.0664856	55	86.24749582	10.81898977
Lactobacillus paracasei	1597	64	98.75731267	176	86.19509982	12.56221286
Lactobacillus sakei	1599	32	97.92944939	16	83.141994	14.78745539
Lactobacillus sanfranciscensis	1625	13	99.50175183	12	79.89737574	19.60437608
Listeria monocytogenes	1639	2293	96.96466429	22	85.44069891	11.52396538
Actinomyces naeslundii	1655	21	96.80905551	55	86.69590357	10.11315194
Bifidobacterium adolescentis	1680	25	98.30018573	334	85.04845242	13.25173331

Bifidobacterium bifidum	1681	31	99.25597	312	85.65199874	13.60397126
Bifidobacterium breve	1685	81	98.80518726	215	87.72513402	11.08005324
Bifidobacterium pseudolongum	1694	19	95.89980164	71	84.92800928	10.97179235
Corynebacterium pseudotuberculosis	1719	77	99.43208418	19	84.99738492	14.43469926
Cutibacterium acnes	1747	137	99.2605673	47	90.70528663	8.555280666
Mycobacterium avium	1764	186	98.82836535	6028	83.38071616	15.44764918
Mycobacterium bovis	1765	97	99.95880591	5865	98.53982125	1.418984665
Mycobacterium fortuitum	1766	21	98.07992026	294	83.10249431	14.97742596
Mycobacterium intracellulare	1767	14	98.69299874	6072	83.19474916	15.49824958
Mycobacterium kansasii	1768	41	97.7989956	5885	83.17072547	14.62827013
Mycobacterium tuberculosis	1773	5354	99.92314808	552	87.47467662	12.44847146
Mycobacterium chelonae	1774	42	95.96206702	1595	84.72284605	11.23922097
Mycobacterium asiaticum	1790	10	93.48028013	5759	82.15862099	11.32165915
Rhodococcus fascians	1828	29	93.06805025	73	87.96471179	5.10333846
Rhodococcus erythropolis	1833	16	97.47887564	34	94.08362243	3.39525321
Streptomyces griseus	1911	21	90.00312271	864	85.15443032	4.848692391
Streptomyces rimosus	1927	32	99.07066714	220	85.33247435	13.73819279
Streptomyces scabiei	1930	15	96.34119924	355	85.59444881	10.74675043
Rothia dentocariosa	2047	10	95.20685654	13	94.635366	0.571490537
Mycoplasma capricolum	2095	11	98.19375687	23	88.44522924	9.748527626
Mycoplasma mycoides	2102	15	96.75717799	19	87.58463622	9.172541769
Ureaplasma urealyticum	2130	13	99.57959337	10	89.69110641	9.88848695
Mesoplasma florum	2151	13	95.95585574	11	86.34890123	9.606954508
Methanosarcina mazei	2209	62	98.94092635	15	85.8497846	13.09114175
Burkholderia mallei	13373	63	99.89846257	1956	91.0525571	8.845905463
Bifidobacterium animalis	28025	38	97.45584656	30	84.9927241	12.46312246
Bifidobacterium pseudocatenulatum	28026	15	98.54906423	199	86.09830802	12.45075621
Staphylococcus lugdunensis	28035	21	99.47445642	320	84.12045968	15.35399675
Streptococcus mitis	28037	57	94.02051013	8319	92.18554634	1.834963791
Lactobacillus curvatus	28038	13	98.83958588	35	83.17237745	15.66720843
Acinetobacter lwoffii	28090	12	91.8148072	157	83.79896404	8.015843156
Burkholderia gladioli	28095	19	98.71760729	1910	84.50961932	14.20798797
Alteromonas macleodii	28108	13	97.21174622	27	84.95612731	12.25561891
Bacteroides ovatus	28116	17	98.02607298	57	88.16044679	9.865626187
Prevotella intermedia	28131	29	96.70939627	12	85.59550678	11.11388949
Cronobacter sakazakii	28141	303	97.89455407	18143	83.18545587	14.7090982
Yersinia kristensenii	28152	18	95.34823481	681	85.45552204	9.892712771
Leptospira noguchii	28182	14	96.54222815	426	87.99518161	8.547046546
Leptospira santarosai	28183	39	98.39702352	402	83.66442423	14.73259929
Leptospira weilii	28184	17	95.20398398	424	84.39074617	10.81323781
Arcobacter butzleri	28197	12	97.90538268	45	81.34765479	16.55772788
Clavibacter michiganensis	28447	23	95.85612228	86	83.29644294	12.55967934
Burkholderia pseudomallei	28450	727	99.36602361	1296	86.7282427	12.63778092
Salmonella enterica	28901	7644	98.84603469	17165	83.13297158	15.7130631
Staphylococcus saprophyticus	29385	44	98.09854312	84	83.41595005	14.68259307
Staphylococcus capitis	29388	40	97.02051353	9637	82.59640776	14.42410577
Acinetobacter haemolyticus	29430	10	96.94959335	2879	82.45229364	14.49729971
Pseudomonas savastanoi	29438	29	98.8424718	1131	86.38091047	12.46156133
Xanthomonas albilineans	29447	15	98.56393382	550	82.83749485	15.72643896
Bradyrhizobium elkanii	29448	11	94.17519795	173	84.81568706	9.35951089
Brucella melitensis	29459	167	99.89762764	381	97.56269647	2.334931163
Brucella suis	29461	53	99.85767992	495	98.05147582	1.806204106
Yersinia frederiksenii	29484	29	89.99037606	671	84.8187365	5.171639562
Yersinia ruckeri	29486	63	99.78926966	640	82.77049347	17.01877619
Vibrio splendidus	29497	68	94.92009849	276	89.11416926	5.805929228
Leptospira kirschneri	29507	28	99.08854757	412	89.01175432	10.07679325
Borreliella garinii	29519	22	98.44455407	120	91.17442042	7.270133648
Mycoplasma ovipneumoniae	29562	12	93.8334791	11	84.50378695	9.329692147
Caldicellulosiruptor bescii	31899	21	99.97613743	12	88.90246646	11.07367098
Cutibacterium avidum	33010	13	98.29661619	174	83.30083878	14.99577741
Yersinia mollaretii	33060	10	96.91955414	689	84.82362905	12.09592509
Pseudomonas viridiflava	33069	12	96.39781628	1100	83.88958798	12.5082283
Rathayibacter rathayi	33887	23	99.61853624	69	89.22202821	10.39650804
Mycobacterium africanum	33894	29	99.95845584	5876	98.72430346	1.234152386
Anoxybacillus flavithermus	33934	10	90.36416796	16	88.62440991	1.739758041
Lactobacillus johnsonii	33959	25	97.37568315	55	86.73618816	10.63949499
Leuconostoc citreum	33964	10	97.53132706	22	82.06307845	15.46824861
Leuconostoc pseudomesenteroides	33968	22	99.7083546	56	85.80249269	13.90586192
Variovorax paradoxus	34073	14	89.73790359	160	83.34007931	6.397824287
Rickettsia japonica	35790	33	99.98925519	89	93.76259356	6.226661631
Bordetella holmesii	35814	28	99.96448244	744	84.5208162	15.44366625
Mycobacterium abscessus	36809	1541	97.8173747	96	85.24539635	12.57197835
Brucella canis	36855	21	99.98717556	527	98.14031584	1.846859717
Enterococcus casseliflavus	37734	15	97.79602078	26	89.85343669	7.94258409
Bartonella henselae	38323	24	99.42714824	86	83.9261735	15.50097474
Acinetobacter johnsonii	40214	11	97.21628609	441	82.87727809	14.339008
Acinetobacter junii	40215	14	97.15091852	2902	82.17012345	14.98079507
Acinetobacter radioresistens	40216	12	98.91183427	151	83.78520544	15.12662883
Stenotrophomonas maltophilia	40324	251	92.70539295	831	82.92840208	9.776990867

Rothia mucilaginosa	43675	19	93.83286968	28	93.98228591	-0.149416228
Rhodococcus hoagii	43767	35	99.17683809	59	82.85418603	16.32265206
Corynebacterium striatum	43770	16	98.45733309	62	85.62389681	12.83343627
Streptococcus cristatus	45634	15	93.2587204	8379	86.33135653	6.927363863
Pseudomonas avellanae	46257	11	98.83429298	631	89.19376033	9.640532654
Lactobacillus rhamnosus	47715	113	98.35755086	128	84.25177953	14.10577134
Lactobacillus crispatus	47770	56	97.90522909	97	83.65456527	14.25066382
Pseudomonas amygdali	47877	55	97.26274349	1306	86.15004143	11.11270206
Serratia fonticola	47917	10	96.24532106	441	83.93325188	12.31206918
Vibrio cyclitrophicus	47951	73	99.1087426	237	88.32990078	10.77884181
Acinetobacter pittii	48296	156	97.15955227	2755	88.20466065	8.95489162
Enterococcus mundtii	53346	15	96.68826803	1000	82.54315054	14.14511748
Lactobacillus lindneri	53444	12	99.92788095	14	80.45386087	19.47402008
Raoultella ornithinolytica	54291	36	99.01520093	14908	83.29038296	15.72481797
Salmonella bongori	54736	11	98.4231935	19217	85.68552539	12.73766811
Vibrio anguillarum	55601	16	98.58077478	736	81.91446462	16.66631017
Xanthomonas arboricola	56448	52	96.46306713	1007	85.99468846	10.46837866
Xanthomonas vasicola	56459	16	98.94629192	860	88.07171788	10.87457404
Citrobacter braakii	57706	19	98.67055833	22873	83.27876513	15.3917932
Burkholderia thailandensis	57975	39	98.92385874	1978	88.64795011	10.27590863
Burkholderia vietnamiensis	60552	40	98.89301796	1986	87.26077964	11.63223832
Enterobacter asburiae	61645	50	96.12178864	11266	83.85981849	12.26197015
Pluralibacter gergoviae	61647	16	98.93204212	15548	82.43906845	16.49297367
Bacillus pseudomycoides	64104	103	97.67731942	2170	85.5314038	12.14591562
Corynebacterium ulcerans	65058	19	97.86144257	77	84.93828835	12.92315421
Pantoea stewartii	66269	10	99.12049789	145	82.60683857	16.51365933
Pseudomonas plecoglossicida	70775	13	91.09330018	3289	82.81056479	8.282735389
Campylobacter lanienae	75658	25	97.58588715	51	87.03684474	10.54904241
Pseudomonas monteilii	76759	12	92.06253815	3169	82.95956204	9.102976104
Serratia plymuthica	82996	11	97.55921797	451	85.62271932	11.93649865
Mycobacterium immunogenum	83262	17	99.79193592	1620	86.7913927	13.00054322
Chlamydia psittaci	83554	60	98.57373377	58	91.24589552	7.327838249
Chlamydia muridarum	83560	26	99.94468033	177	81.57440455	18.37027578
Achromobacter xylosoxidans	85698	35	95.58089481	828	85.28256046	10.29833434
Pseudomonas lundensis	86185	20	97.96861458	406	83.27646443	14.69215014
Burkholderia multivorans	87883	86	97.85677513	1951	87.25636114	10.60041399
Xanthomonas gardneri	90270	12	99.79757182	1020	86.00557942	13.7919924
Burkholderia cenocepacia	95486	255	98.74198204	1775	87.44650704	11.295475
Bordetella hinzii	103855	10	99.63923798	883	83.87717176	15.76206622
Acinetobacter nosocomialis	106654	39	97.87501727	2849	91.04430275	6.830714519
Acinetobacter ursingii	108980	10	98.18374337	2916	81.40498022	16.77876315
Porphyromonas gulae	111105	11	98.29877472	61	92.64533359	5.653441132
Lactobacillus parabuchneri	152331	23	98.89362335	15	81.70784454	17.18577881
Burkholderia ambifaria	152480	10	96.40345001	2016	87.54503664	8.858413376
Bacillus toyonensis	155322	200	99.14165817	2075	91.18544421	7.956213962
Enterobacter hormaechei	158836	354	96.25438302	24933	83.03709788	13.21728514
Elizabethkingia miricola	172045	13	96.58816294	96	90.29884291	6.289320029
Enterovibrio norvegicus	188144	10	92.57033234	13	85.40616842	7.164163912
Paenibacillus odorifer	189426	31	97.03072283	24	83.23614724	13.79457559
Vibrio coralliilyticus	190893	13	97.05618873	300	82.95961095	14.09657778
Dickeya zeae	204042	10	96.36544876	87	83.72069786	12.6447509
Enterobacter kobei	208224	29	88.00185022	19789	82.68026521	5.321585005
Escherichia albertii	208962	48	98.82006613	19175	87.47205149	11.34801465
Vibrio tasmaniensis	212663	11	94.43620925	307	89.30882676	5.127382488
Bifidobacterium longum	216816	111	97.25482186	218	87.14281836	10.1120035
Phaeobacter inhibens	221822	21	97.68718792	41	85.91968108	11.76750684
Mycobacterium chimaera	222805	19	99.46958457	5908	83.00251552	16.46706905
Pseudomonas psychrotolerans	237610	16	90.77412748	2626	82.74163635	8.032491136
Klebsiella variicola	244366	66	99.05228875	15455	85.69264321	13.35964554
Vibrio crassostreae	246167	14	95.63223158	334	88.34193854	7.290293032
Streptococcus pseudopneumoniae	257758	36	93.14407174	8323	94.65339374	-1.509322001
Bacillus halotolerans	260554	15	98.49594116	401	84.11207344	14.38386773
Staphylococcus pseudintermedius	283734	88	99.39119608	35	89.28104401	10.11015207
Bacillus altitudinis	293387	11	98.29882164	110	92.09970935	6.199112285
Enterobacter ludwigii	299767	12	98.0426608	16711	82.90557647	15.13708433
Alteromonas mediterranea	314275	11	98.10163741	28	84.04326657	14.05837084
Streptococcus gallolyticus	315405	19	98.18162737	50	88.01926147	10.1623659
Mycobacterium colombiense	339268	18	93.68381141	6116	82.89935817	10.78445324
Salinispora pacifica	351187	37	94.57629601	123	86.12226477	8.454031238
Xanthomonas fuscans	366648	29	99.65428761	988	88.73473498	10.91955263
Fischerella thermalis	372787	29	99.11745216	13	90.84017534	8.277276826
Pseudomonas protegens	380021	18	97.96502304	3877	82.73607414	15.22894891
Bacillus aryabhattai	412384	17	96.03637351	99	95.11844591	0.917927597
Cronobacter dublinensis	413497	30	98.03331967	10545	83.59336895	14.43995072
Cronobacter malonaticus	413503	40	98.96979396	647	88.99396632	9.975827636
Xanthomonas perforans	442694	33	99.90954104	1013	88.21741211	11.69212893
Mycobacterium conceptionense	451644	11	98.97508857	346	83.32269203	15.65239654
Xanthomonas euvesicatoria	456327	37	99.79539222	1007	88.236548	11.55884422
Burkholderia contaminans	488447	10	98.17966588	2006	87.74488343	10.43478245

Bacillus velezensis	492670	106	98.33685479	308	85.27599981	13.06085498
Lactobacillus taiwanensis	508451	25	98.94200378	55	86.88863331	12.05337047
Bacillus safensis	561879	19	97.57023982	101	91.22619319	6.344046628
Bacillus cytotoxicus	580165	13	99.62997202	2261	84.289633	15.34033902
Pseudomonas chlororaphis	587753	28	95.03477878	4321	82.86837278	12.166406
Vibrio owensii	696485	13	96.86378193	1325	83.75501724	13.10876469
Acinetobacter indicus	756892	30	97.42424077	175	82.91518643	14.50905434
Vibrio jasicida	766224	16	98.08083153	1200	83.4333204	14.64751113
Staphylococcus argenteus	985002	111	99.01716249	9346	87.977647	11.0395155
Staphylococcus agnetis	985762	10	97.38688736	12	82.52060827	14.86627909
Dickeya solani	1089444	18	99.05554132	411	83.03753025	16.01801106
Elizabethkingia anophelis	1117645	60	98.23889066	50	87.93544308	10.30344759
Klebsiella michiganensis	1134687	63	97.27808634	4273	84.98728944	12.2907969
Gilliamella apicola	1196095	68	88.53862	24	83.12045097	5.418169022
Enterobacter xiangfangensis	1296536	27	98.47499198	23711	83.1585587	15.31643327
Klebsiella quasipneumoniae	1463165	137	97.93810154	13112	86.23997177	11.69812977
Vibrio metoecus	1481663	18	97.3682891	760	87.27402744	10.09426166
Bacillus paralicheniformis	1648923	14	98.80758013	91	93.55074671	5.256833422
Bacillus wiedmannii	1890302	135	96.68626907	2140	91.68330614	5.002962932
Xanthomonas phaseoli	1985254	93	98.04736471	954	87.50532241	10.5420423
